# Marginal bone loss around platform-switched and non-platform switched implants after two years of placement: a clinical trial

**DOI:** 10.15171/joddd.2017.005

**Published:** 2017-03-15

**Authors:** Ali Zarandi, Maryam Novin

**Affiliations:** ^1^Assistant Professor, Department of Periodontics, Faculty of Dentistry, Tabriz University of Medical Sciences, Tabriz, Iran; ^2^Private Practice, Tabriz, Iran

**Keywords:** Bone resorption, dental implant, periapical

## Abstract

***Background.*** The present study was conducted to investigate the marginal bone loss around two different types of implant‒abutment junctions, called platform-switched (Implantium system) and non-platform switched (XiVE system) after two years of loading.

***Methods.*** Sixty-four implants in 49 patients were included in the study. The implants were placed in the posterior mandibular region according to the relevant protocols. The extent of bone loss around the implants was measured and compared after 24 months, using digital parallel periapical radiographs.

***Results.*** The means ± SE of bone loss values in the platform-switched and non-platform-switched groups were 0.47 ± 0.048 mm and 1.87 ± 0.124 mm, respectively. The difference between the two groups was statistically significant (P < 0.0001).

***Conclusion***. The platform-switching technique seems to reduce the periimplant crestal bone resorption, which supports the long-term predictability of implant therapy.

## Introduction

The aim of dental implantology is to preserve the peri-implant tissues in long term.^1,2^ It has been revealed that bone remodeling after dental implant loading would affect bone loss.^3,4^ The etiology of these bone changes has not been entirely explained. However, factors including trauma to the bone and periosteum after surgery, deficiency of biomechanical stability under pressure, size of microgaps between the implant and the abutment, and bacterial colonization in the implant groove may affect bone changes.^1-6^

Nevertheless, a small bone loss after implant loading would not have an undesirable impact on the success of the implant. The estimation of alterations in the size of crestal bone adjacent to the implant is considered to be the standard criterion for the evaluation of implant outcome.^7^ Presently, many researchers advocate to consider the preservation of marginal bone, surface specifications of implants, the diameter of implant and its loading depth, increased microthreads, use of single implants and platform-switching systems.^8^

The platform-switching technique was initially utilized in the mid-1980s. Previously, implants with large diameters were applied; however, such abutments were not accessible. Thus, thinner abutments were needed. However, the level of vertical crestal bone loss was less than what was expected in this method in the long term.^9^ This might be related to the increased distance between the alveolar crest and the implant‒abutment border. The benefits of the platform system are reduction in mechanical pressures on the crestal bone, location of the papilla on the bony ring, and facilitation of blood stream in the bone, mainly when the distance decreases between the implants. The platform system may also decrease the risk of bone loss in comparison with the conventional implants.^10-12^

Although this system has many advantages, more research is necessary to assess its clinical success. Many of the previous studies have been undertaken 3‒12 month after surgery^13-15^, and the investigation of implants 24 months after implant placement is scarce. Therefore, the aim of the present study was to assess the amount of bone loss around the platform-switched and non-platform-switched implants 24 months after placement of implants.

## Methods

This study was registered in the Iranian Registry of Clinical Trials (code: TBZMED.REC.1394.442). The patients were selected from the private practice of the first author based on the following inclusion criteria: patients aged 25‒67 years old without diabetes and osteoporosis, no smoking and alcohol use, no bleeding disorders, no intake of immunosuppressive drugs and those targeting bone metabolism such as bisphosphonates, no implants with bridge, no bruxism, no use of bone grafts for the dental implants, no periapical lesions, no tooth fenestration and alveolar bone infection.

In the present clinical trial, 32 platform-switched (Implantium system) and 32 non-platform-switched (XiVE system) implants were placed posterior mandibular region according to the relevant protocols in the. The implants were placed at the same level with crestal bone in the platform-switched technique, and at the crest‏ module in the non-platform technique.

One technician took standardized parallel periapical radiographs of the implants after 2 years of placement. The marginal bone loss was measured from the radiographs by Vernier caliper in the two groups. Each distance was measured by two examiners, on two occasions with a 1-month interval between the two measurements. Intra- and inter-examiner reliability was determined using intra-class correlation coefficients (ICCs).^16,17^

The data are presented as means ± SE and were analyzed by SPSS 17 at a significance level of P ≤ 0.05. ANOVA was applied to evaluate the effect of implant type on bone loss variable.^18,19^

## Results

**Figure 1.**
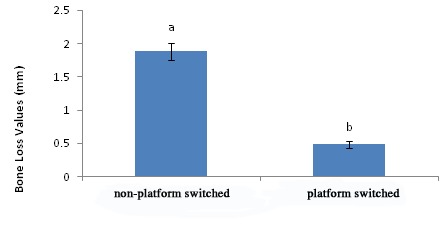
Results of bone loss in platform- and non-platform-switched implants (Values represent the mean value of experiments performed in 32 implant ± SE).

Figure 1 illustrates the effect of two implant types (platform-switched and non-platform-switched) on bone loss. Mean marginal bone loss was 0.47 ± 0.048 mm for platform- and 1.87 ± 0.124 mm for non-platform-switched implants. The difference between the two groups was statically significant (P < 0.0001) (Table 1).

**Table 1:** Analysis of variance (ANOVA) for bone loss in two implant types (platform- and non-platform switched)

**Source**	**Type III Sum of Squares**	**df**	**Mean Square**	**F**	**Sig.**
**Corrected Model**	31.402a	1	31.402	109.423	0
**Intercept**	88.666	1	88.666	308.962	0
**Type of implant**	31.402	1	31.402	109.423	0
**Error**	17.793	62	0.287		
**Total**	137.861	64			
**Corrected Total**	49.195	63			

Dependent Variable: bone loss

## Discussion

Completely or partially edentulous patients do not have the capacity for normal functions such as chewing and speaking. Hence, the aim of modern dentistry is the restoration of oral health and good appearance. Moreover, other aspects of social function may also be affected by losing aesthetics and beauty of the patient.^20^ Removable partial dentures or full dentures are not perfectly comfortable for patients and the patients mostly prefer fixed prostheses such as implants.

In the present study, the marginal bone loss in the platform- and non-platform-switched implants was investigated. The marginal bone loss was significantly lower in the platform-switched implants compared to the non-platform-switched implants. The limitation of the study was its relatively small sample size.

In this line, Wang et al^15^ achieved similar results after 1 year.Their result is the same as our study but the differences are related to time of placement of the final restoration and the fact that bone loss was estimated after 1 year. Earlier studies have shown that maximum bone loss occurs during the first year after loading the implants.

In a clinical and radiographic prospective study, Cappiello et al^21^ assessed bone loss around platform-switched and non-platform-switched implants. Their results confirmed the main role of the microgap between the implant and abutment in the alteration of the peri-implant crestal bone. Platform switching appears to decrease the peri-implant crestal bone resorption.^21^ Similarly, Almedia et al^22^ concluded that the replacement of the implant‒abutment microgap in platform switching with Frialit-2 system implants might be effective in reducing marginal bone loss.

Vigolo et al^23^ observed that marginal bone loss at the second, third, fourth, and fifth years after abutment and crown insertion did not exhibit any significant variations. Canullo et al^3^ proposed that marginal bone level changes could be related to the amount of implant/abutment mismatching. In their study, marginal bone level was better preserved in implants restored by platform-switching implants.^3^ Tramel et al^24^ proposed that the insertion of a more medialized abutment on platform-switching implants might decrease bone loss. Their results are similar to our results, suggesting that less crestal bone loss happens around a platform-switched implant against a conventional implant

In addition, Kapoor et al^13^ observed that bone loss value in platform-switched implants was not significantly different between the mesial and distal sides of crestal bone. In another study, no significant differences were observed between the two types of platform-switched implants (Nobel Active and Nobel Replace Groovy).^14^

## Conclusion

The platform-switched implants exhibited significantly less bone loss in comparison with the non-platform switched implants after 2 years of placement. It can be concluded that the platform-switched implants can be successfully used. However, further studies are recommended in this regard.

## Acknowledgments

The authors would like to thank the staff in the Department of Periodontics.

## Authors’ contributions

A Z. designed the study and M N.carried out the study procedures. Also A Z. carried out the statistical analyses, explanation of data andpreparing the draft of manuscript. All the authorscontributed to the final draft, read and approved the finalmanuscript.

## Funding

For this study, no funding was obtained.

## Competing interests

The authors declare that they have no conflict of interest.

## Ethics approval

The study was approved by the Research EthicsCommittee of Tabriz University of Medical Sciences (No. 9278) andregistered in the Iranian Registry of Clinical Trials (code: TBZMED.REC.1394.442).
